# Divergent pattern of functional connectivity within the dorsal attention network differentiates schizophrenia and bipolar disorder patients

**DOI:** 10.3389/fpsyt.2024.1474313

**Published:** 2024-09-19

**Authors:** Adrian Andrzej Chrobak, Sylwia Bielak, Dominik Nowaczek, Aleksandra Żyrkowska, Anna Maria Sobczak, Magdalena Fafrowicz, Amira Bryll, Tadeusz Marek, Dominika Dudek, Marcin Siwek

**Affiliations:** ^1^ Department of Adult Psychiatry, Jagiellonian University Medical College, Kraków, Poland; ^2^ Department of Adult, Child and Adolescent Psychiatry, University Hospital in Cracow, Kraków, Poland; ^3^ J. Dietl Specialist Hospital, Kraków, Poland; ^4^ Department of Cognitive Neuroscience and Neuroergonomics, Institute of Applied Psychology, Jagiellonian University, Kraków, Poland; ^5^ Doctoral School in the Social Sciences, Jagiellonian University, Kraków, Poland; ^6^ Chair of Radiology, Jagiellonian University Medical College, Kraków, Poland; ^7^ Faculty of Psychology, SWPS University, Katowice, Poland; ^8^ Department of Affective Disorders, Jagiellonian University Medical College, Kraków, Poland

**Keywords:** parietal cortex, frontal eye fields, resting state, functional networks, schizophrenia, bipolar disorder

## Abstract

**Introduction:**

Schizophrenia (SZ) and bipolar disorder (BD) share common clinical features, symptoms, and neurocognitive deficits, which results in common misdiagnosis. Recently, it has been suggested that alterations within brain networks associated with perceptual organization yield potential to distinguish SZ and BD individuals. The aim of our study was to evaluate whether functional connectivity (FC) of the dorsal attention network (DAN) may differentiate both conditions

**Methods:**

The study involved 90 participants: 30 remitted SZ patients, 30 euthymic BD patients, and 30 healthy controls (HC). Resting state functional magnetic resonance imaging was used to compare the groups in terms of the FC within the core nodes of the DAN involving frontal eye fields (FEF) and intraparietal sulcus (IPS)

**Results:**

BD patients presented weaker inter-hemispheric FC between right and left FEF than HC. While SZ did not differ from HC in terms of inter-FEF connectivity, they presented increased inter- and intra-hemispheric FC between FEF and IPS. When compared with BD, SZ patients showed increased FC between right FEF and other nodes of the network (bilateral IPS and left FEF)

**Conclusion:**

We have shown that altered resting state FC within DAN differentiates BD, SZ, and HC groups. Divergent pattern of FC within DAN, consisting of hypoconnectivity in BD and hyperconnectivity in SZ, might yield a candidate biomarker for differential diagnosis between both conditions. More highly powered studies are needed to confirm these possibilities

## 1 Introduction

Schizophrenia (SZ) and bipolar disorder (BD) are chronic and severe mental disorders that presents significant overlap in their clinical features (1), cognitive dysfunctions (2), neurological deficits (3–5), as well as structural and functional brain abnormalities (6). Due to the similarities, both conditions are often misdiagnosed (1). Chart records show that SZ is the second most common misdiagnosis of BD (7), and up to 31% of those patients may be diagnosed with SZ or other psychotic disorder (8). Thus, there is a vital need to seek for the objective biomarkers that can differentiate between these two conditions.

With that aim, a growing number of studies use functional magnetic resonance imaging (fMRI), a promising and non-invasive method that can capture the intrinsic architecture of brain activity alterations in SZ and BD (9–11). This technique enables evaluation of large-scale functional networks, distributed sets of brain areas synchronically activated at rest or during task performance that corresponds with cognitive and mental processes (12). Those systems include default mode network (DMN), salience network (SAN), limbic network (LIN), frontoparietal network (FPN), somatosensory network (SMN), visual network (VIN), and dorsal attention network (DAN) (12). Recently, the latter one has become an object of interest due to its potential to differentiate SZ and BD patient groups (13). The DAN is recruited when attention is voluntarily shifted to spatial locations or during intentional visual exploration using eye movements (14). This network is composed of bilateral regions in the parietal and frontal cortex involving the intraparietal sulcus (IPS), which plays a key role in the selection among competing stimuli, and the frontal eye fields (FEF), which contain a representation of the priority of items in the visual field, to plan and execute eye movements (15). Recently, Keane et al. (2023) analyzed the activity of this network showing that among 12 large-scale functional networks, only the DAN was capable of differentiating BD and SZ during perceptual organization task (13). Authors suggested that altered activity within this functional network might yield a candidate biomarker for differential diagnosis of those conditions (13).

Neuroimaging and neurophysiological studies report alterations within DAN structures in both conditions. SZ patients have been shown to present oculomotor deficits that are associated with disrupted activity of the FEF (16–18) and parietal cortex (18). Moreover, increased activity within parietal regions of DAN as well as FEF hypoactivation has been linked with working memory impairments in this clinical group (19, 20). rs-fMRI and recent EEG studies demonstrated that SZ patients present DAN dysconnectivity that may be related to disruptions in attentional processes and integration of information from a different brain area (21–23). Only a few studies evaluated dysfunctions of this network in BD. Two rs-fMRI analyses demonstrated that euthymic BD patients present altered FEF activity and a decreased functional connectivity (FC) with thalamus and cerebellar structures related to oculomotor control (24, 25). A single fMRI study has shown that hypoactivation of this region is associated with working memory deficits in this clinical group (20). Studies comparing both clinical groups demonstrated that contrary to BD, SZ individuals present altered brain responses during attentional processes (26, 27). These observations encourage further studies on DAN activity in those conditions. Despite the popularity of fMRI analyses in psychiatric disorders, there is scarcity of studies directly comparing the activity of the large-scale functional networks between SZ and BD patients. Interestingly, most of the single scanner studies did not include DAN in their analyses (28–33). Majority of research on functional connectivity (FC) in SZ and BD perform whole-brain analyses rather than hypothesis-driven evaluation of specific networks resulting in substantial variety of findings and leading to contradictory conclusions (34). To the best of our knowledge, no study has performed hypothesis-driven comparisons of DAN FC in these disorders.

Resting state fMRI (rs-fMRI) evaluates baseline neuronal brain activity during the absence of goal-directed external stimuli. An important advantage of this method is the lack of behavioral task performed by the participants, which significantly simplifies the procedure, shortens scanning time, and minimizes its burden on patients. While Kaene et al. (2023) showed that DAN activity during perceptual organization task may differentiate the BD and SZ groups (13), in our study, we aimed to test whether FC of this network measured during resting state may also reveal such potential. If alterations in resting state FC (rs-FC) within DAN are sufficient to differentiate the abovementioned groups, the use of the rs-fMRI would significantly facilitate application of this biomarker in clinical practice. We hypothesize that altered FC of this large-scale network may differentiate BD, SZ, and healthy individuals.

## 2 Methods

### 2.1 Participants

The study was conducted on a group of participants described in our previous research (3). They were 30 SZ and 30 BD patients (12 BD I and 18 BD II patients), and 30 healthy controls (HCs). The diagnosis and clinical evaluation were performed by an experienced psychiatrist according to DSM-5 and ICD-10. The inclusion criteria for the BD group were the state of euthymia defined as <11 points in the Montgomery–Åsberg Depression Rating Scale (35) and <5 points in the Young Mania Rating Scale (36). SZ patients were recruited in the state of symptomatic remission, defined as three or fewer points on all Positive and Negative Syndrome Scale items. In our study, we focused on patients during a state of symptomatic remission as we aimed to assess FC alterations related to the diagnosis (trait marker) rather than those associated with the presence of affective or psychotic episode (state marker). Both clinical groups were treated with neuroleptics from the group of dibenzoxazepines (quetiapine, olanzapine, or clozapine). The selection of the abovementioned drugs provided a relative pharmacological homogeneity across patient groups. Patients undertaking lithium were excluded as the treatment may have had an impact on patients’ motor performance and cerebellar functions, which were evaluated in our project (3). Valproic acid and lamotrigine treatment was allowed. Exclusion criteria involved the following: history of drug or alcohol abuse according to the substance use disorder of DSM-5; chronic, severe, or acute somatic diseases; severe personality disorders; and treatment other than those mentioned in the inclusion criteria. HCs were recruited from the group of all of the authors’ social network (family members, friends, friends’ family members, as well as their acquaintances), and they were evaluated by an experienced psychiatrist. Healthy volunteers represented a wide range of communities and were not restricted to a single institution (e.g., University or Hospital). All of the individuals in this group reported a negative history of neurological and mental disorders and did not meet any exclusion criteria for patients. The SZ, BD, and HC groups were matched in terms of age and gender. Patient groups were matched for duration of their treatment. All of the participants were right-handed as measured by the Neurological Evaluation Scale (37). **Table 1** presents the demographic characteristics of the studied groups. The study was approved by the Jagiellonian University Bioethic Committee. All participants signed a written consent prior to the assessment.

**Table 1:** Description of examined groups.

Groups	SZ	BD	HC
Age [mean (SD)]^a^	40.57 (12.38)	38.3 (11.88)	39.73 (11.63)
Sex (men/women^)b^	12/18	18/12	16/14
Years of education [mean (SD)]^c^	15.3 (2.67)	16.27 (2.68)	15.33 (2.12)
Duration of treatment^d^	10.47 (6.75)	8.2 (6.98)	–
Equivalent of olanzapine daily dosage [mg/day, (SD)]^e^	11.79 (6.1)	10.17 (5.3)	–
BD type (I/II)	–	13/17	–

There were no statistically significant differences between studied groups in terms of the abovementioned parameters.

^a^F(2,87) = 0.275, p = 0.76. ^b^χ2(2.90) = 2.49, p = 0.28. ^c^F(2,87) = 1.442, p = 0.24. ^d^T(58) = −1.279, p = 0.21. ^e^T(58) = 0.182, p = 0.86.

SZ, schizophrenia; BD, bipolar disorder; HC, healthy controls; SD, standard deviation.

### 2.2 MRI acquisition

The procedure of MRI acquisition was adopted from our previous study (38). Siemens Skyra MR System (Siemens Medical Solutions, Erlangen, Germany) was used to acquire MRI data. Anatomical images were obtained using sagittal 3D, T1-weighted MPRAGE sequence with TR = 1,800 ms, TE = 2.26 ms, TI = 900 ms, voxel size of 1 mm^3^, and with the use of 64 channels coil. The total number of slices in the sagittal plane equaled 208, FOV = 256 × 256 mm^2^. Whole-brain images were covered with 20% gap axial slices and a distant factor of 0.5 mm. Generalized Autocalibrating Partial Parallel Acquisition (GRAPPA) with a factor of 3 was used to reduce the imaging time. A total of 13 min and 20 s of rs-fMRI EPI images were acquired using gradient-echo single-shot echo planar imaging sequence with the following parameters: TR = 800 ms; TE = 27 ms; slice thickness = 0.8 mm, voxel size = 3 mm^3^, and with no gap using 64-channel coil. A total of 52 inter leaved transverse slices and 1,000 volumes were obtained. Participants were instructed to keep their eyes open and not to think about anything during the scanning procedure. Simultaneous-multi-slice acquisition was acquired to enhance the sensitivity of hemodynamic response resulting in reduction of TR to 0.8 s.

### 2.3 Imaging data preprocessing

The rs-fMRI data processing was performed with the use of Data Processing & Analysis for Brain Imaging (DPABI) V6.0 (39) as well as SPM 12 (Wellcome Trust Centre for Neuroimaging, UCL, London, United Kingdom) in MATLAB version R2018a (The MathWorks, Inc., Natick, MA, United States). Preprocessing consisted of the following steps: 1) conversion of data from DICOM to Nifti format; 2) removing the first 10 time points to eliminate the effect of magnetic field instability; 3) slice timing correction, which is responsible for synchronizing the image that was originally collected at different time points. Image timing correction has been shown to reliably increase the sensitivity and effect power; 4) realignment—data on head movements were extracted and processed for each subject. The maximum deviation criterion was <3 mm or <3^o^; 5) voxel specific head motion—analysis and detection of head movements with an accuracy of 1–2 voxels (the smallest brain measurement units, 2 mm × 2 mm × 2 mm); 6) coregistration Fun-T1—the data of each subject were checked for matching functional (EPI) to structural data (T1) based on anatomical landmarks (brain ventricles and border points); 7) cropping and reorienting T1 images—cropping and transforming the image structural; 8) T1 Segmentation + DARTEL brain segmentation and normalization; 9) T1 coregistration to Fun—unification of all time points (1,000 points in time) so that each patient has one map of brain activity; 10) normalization using EPI template to the MNI space; 11) covariate regression: removing signal from white matter and cerebrospinal fluid so that only signal from gray matter remains; 12) CompCor—principal component analysis (PCA) of the first five components. PCA is a technique for reducing the dimensionality of such data sets, which minimizes information loss and increasing interpretability of the results.

### 2.4 Functional connectivity analysis

Region of interest (ROI)-to-ROI FC analysis was performed to evaluate the resting state activity with the DAN. ROIs were chosen based on the Harvard–Oxford Atlas and localized regarding their coordinates on the x-, y-, and z-axes. Raw time courses were extracted from each subject using “ROI Signal Extractor” module in Data Processing & Analysis for Brain Imaging (DPABI) V4.3 (39) in MATLAB version R2018a and SPM 12. The DAN was defined with the following ROIs: right FEF [FEF(R), 30, −6, 64], left FEF [FEF(L), −27, −9, 64], right IPS [IPS(R), 39, −42, 54], and left IPS [IPS(L), −39, −43, 52].

### 2.5 Statistical analysis

Demographic characteristics were compared with the use of one-way ANOVA, t-tests, and χ2 tests as appropriate. The Levene test was used to evaluate the homogeneity of variance. These data were reported by Chrobak et al. (2023) (3). One-way ANOVA corrected with the use of Benjamini and Hochberg (1995) false discovery rate (FDR, p < 0.05) was applied to compare FC among ROIs within the DAN between the SZ, BD, and HC groups (40). Bonferroni *post-hoc* tests were applied to perform pairwise comparisons. Antipsychotics’ daily dosage was converted to the equivalent of olanzapine according to Leucht et al. (2015) (41). Associations between demographic and clinical variables (age, duration of the treatment, years of education, and the equivalent of the daily dose of olanzapine) and FC measures were evaluated using Pearson correlations. Statistical analyses were made in R software (42).

## 3 Results

**Figure 1:**
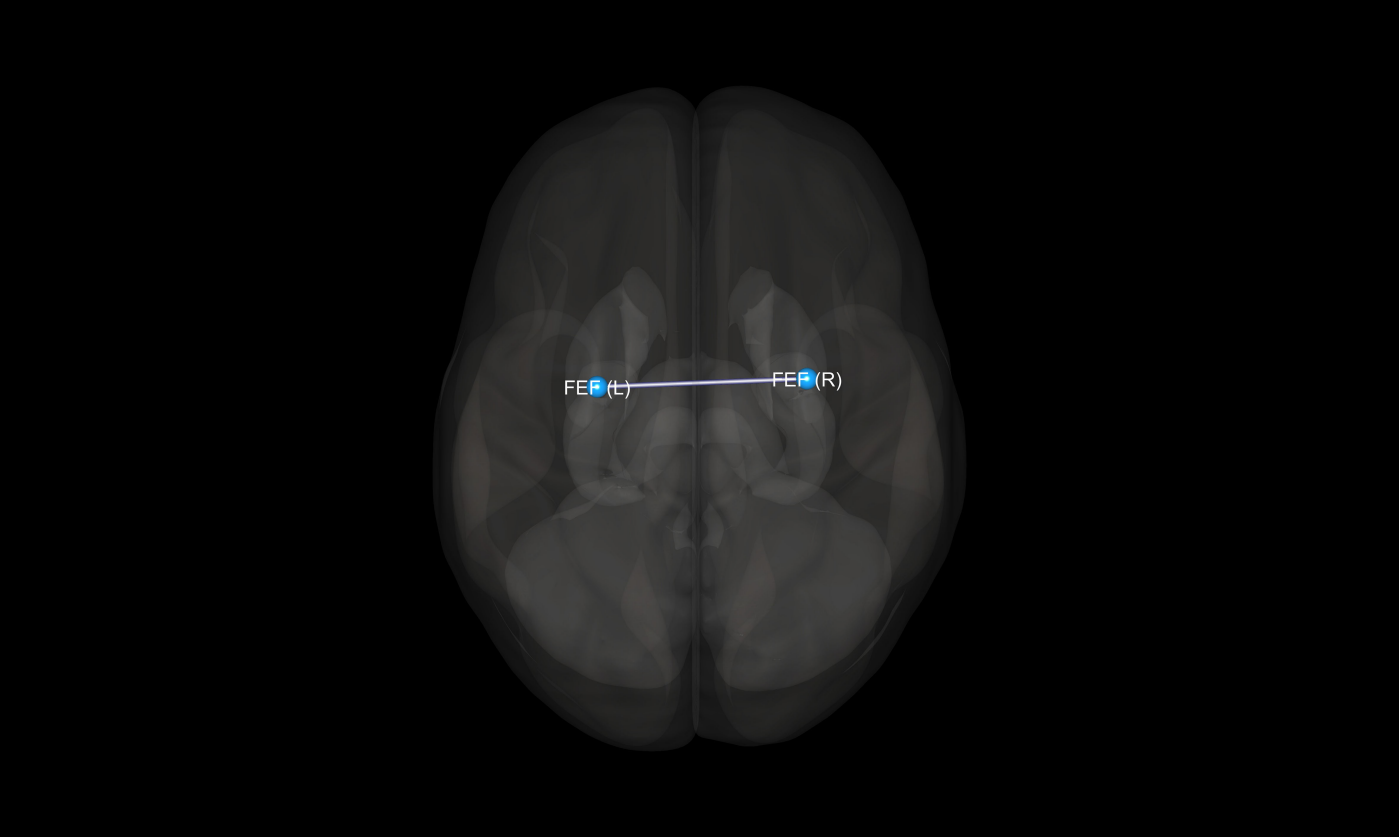
Visualization of significant differences in FC between bipolar disorder and healthy controls groups. FEF, frontal eye fields; (R), right; (L), left.

**Figure 2:**
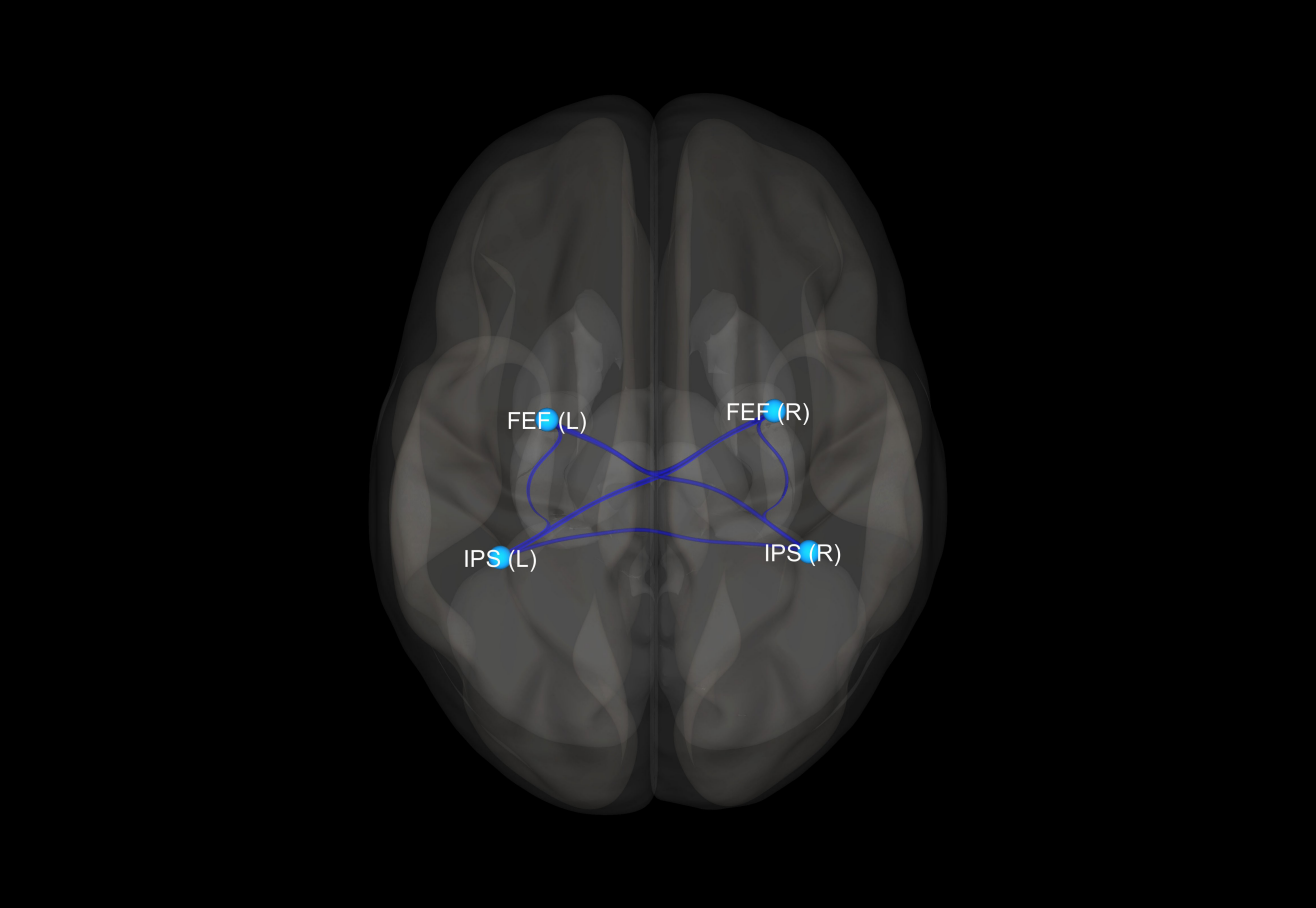
Visualization of significant differences in FC between schizophrenia and healthy control groups. FEF, frontal eye fields; IPS, intraparietal sulcus; (R), right; (L), left.

**Figure 3:**
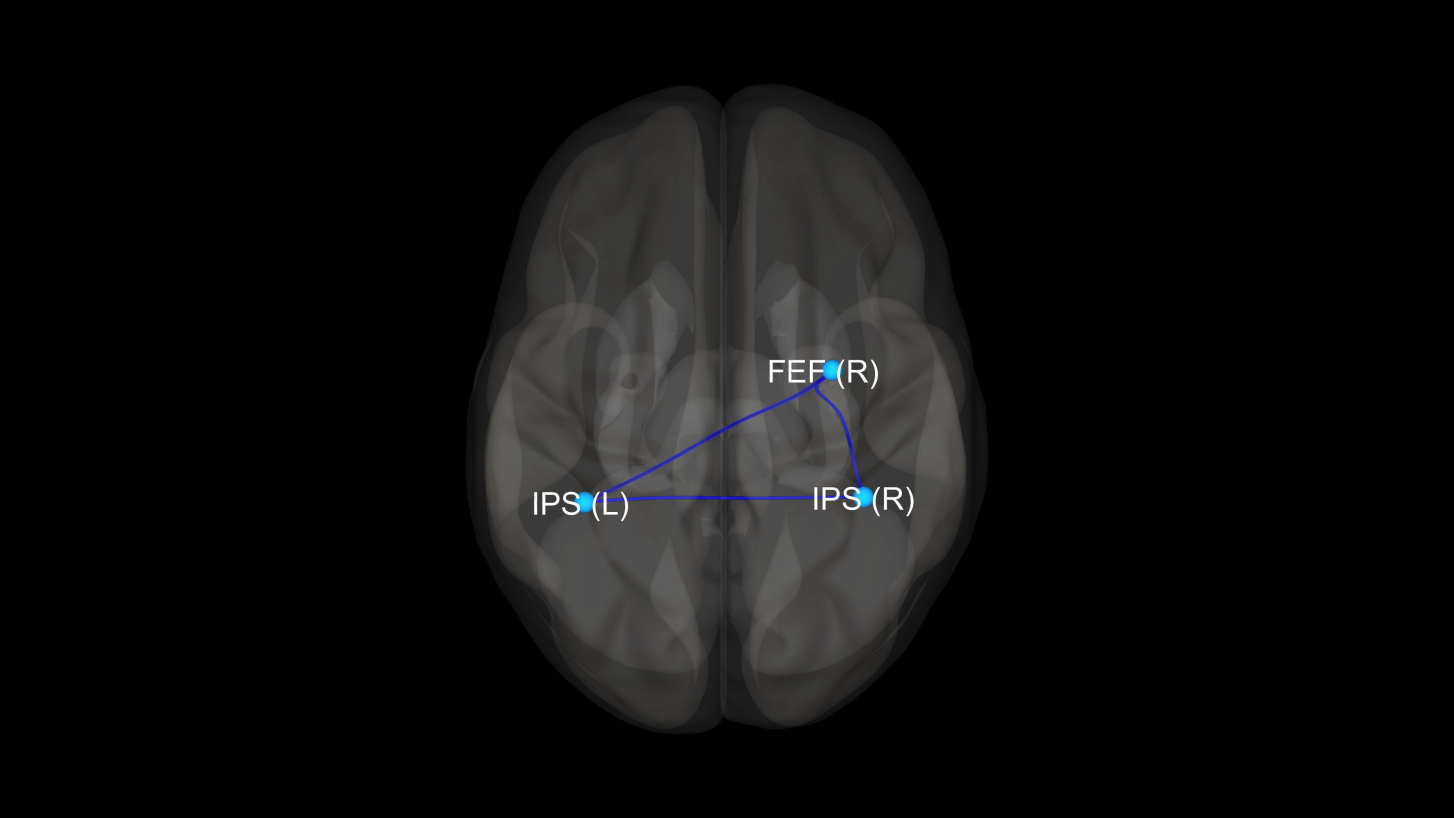
Visualization of significant differences in FC between schizophrenia and bipolar disorder patient groups. FEF, frontal eye fields; IPS, intraparietal sulcus; (R), right; (L), left.

**Figure 4:**
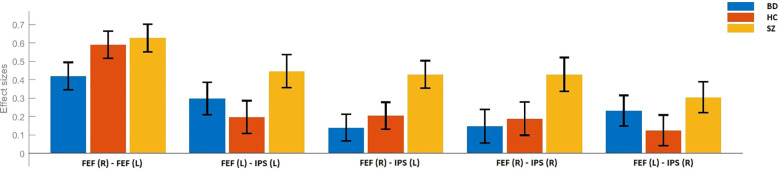
Effect sizes for the differences in FC between schizophrenia (SZ), bipolar disorder (BD), and healthy control (HC) groups. FEF, frontal eye fields; IPS, intraparietal sulcus; (R), right; (L), left.

There were no statistically significant differences between the studied groups in terms of age, sex, years of education, duration of treatment, and equivalent of olanzapine daily dosage. Detailed results of those comparisons are presented in **Table 1**. There were significant differences between the SZ, BD, and HC groups in FC measures between the following ROI–ROI pairs: FEF(R)–FEF(L), FEF(R)–IPS(R), FEF(R)–IPS(L), and FEF(L)–IPS(L). In comparison to HC, BD patients presented decreased FC between FEF(R) and FEF(L). The SZ group showed stronger FC between FEF(R)–IPS(R), FEF(R)–IPS(L), FEF(L)–IPS(L) than HC. Finally, in comparison to BD, SZ patients revealed increased FC between FEF(R)–FEF(L), FEF(R)–IPS(R), and FEF(R)–IPS(L). Detailed results of those comparisons are presented in **Table 2**. **Figures 1**–**3** depict networks revealing significant differences between the studied groups. **Figure 4** presents the effect sizes, which reflect the strength of FC between the ROIs compared between the groups. There were no statistically significant correlations between FC measures and participant age (p = 0.08), years of education (p = 0.12), duration of treatment (p = 0.07), and the daily dose of olanzapine (p = 0.5).

**Table 2:** Functional connectivity differences among schizophrenia (SZ), bipolar disorder (BD), and healthy controls (HC) groups.

Region	F(2,86)	p (corrected)	Pairwise comparisons
FEF(R)–FEF(L)	6.06	0.007	BD < HC (p = 0.03) BD < SZ (p = 0.007)
FEF(R)–IPS(R)	7.6	0.003	HC < SZ (p = 0.006) BD < SZ (p = 0.003)
FEF(R)–IPS(L)	11.67	<0.001	HC < SZ (p = 0.006) BD < SZ (p = 0.0002)
FEF(L)–IPS(L)	5.45	0.009	HC < SZ (p = 0.003)

Statistical differences were computed using one-way ANOVA with false discovery rate correction (p < 0.05).

FEF, frontal eye fields; IPS, intraparietal sulcus; (R), right; (L), left.

## 4 Discussion

Our results have shown that altered resting state FC within DAN differentiates the BD, SZ, and HC groups. In comparison with HC, BD patients revealed inter-hemispheric hypoconnectivity between FEF regions. While the SZ group did not differ from HC in terms of these connections, they presented significant inter- and intra-hemispheric hyperconnectivity between FEF and IPS [FEF(R)–IPS(R), FEF(L)–IPS(L), and FEF(R)–IPS(L)]. Finally, according to our hypothesis, FC within DAN differentiated both clinical groups. In comparison with BD, SZ patients presented increased FC between FEF(R) and other nodes of this network [IPS(L), IPS(R), and FEF(L)].

It is hypothesized that interhemispheric functional connections between FEF regions are responsible for achieving consensus on the destination of the next saccade. In such system, cells that command similar movements mutually excite each other while inhibiting those that would produce conflicting movements, thus resolving the conflict through winner-takes-it-all mechanism (43). Machner et al. (2022) have shown that in the neurotypical brain, increased FC between FEFs is associated with improved learning (shorter reaction times) during orienting/reorienting task (14). Our results indicating lower FC among FEF in BD correspond with the observations of oculomotor deficiencies in BD including decreased ratio of eye velocity to target velocity, higher number of saccades, and vergence eye movement impairments (44, 45). Decreased FC in BD within DAN has been also linked with the presence of euthymia (46). Brady et al. (2017) have evaluated the group of 23 manic and 24 euthymic BD I patients, and 23 HC, indicating that FC between frontal, parietal, and occipital nodes within the DAN are significantly greater in mania than euthymia or HC subjects (46). However, BD patients in euthymia presented lower FC within DAN than HC, which is congruent with our study, strengthening the results. Further neuroimaging studies are necessary to identify the impact of the abovementioned factors on FC within DAN in BD patients across different affective episodes. Notably, BD patients do not differ from HC in FC associated with bilateral IPS indicating that BD patients do not exhibit significant impairments in visuospatial working memory, which involves recalling and manipulating images while remaining oriented in space (47). Surprisingly, the intrinsic architecture of brain activity in BD patients resembles HC participants more than SZ, while the FC between BD and HC remains significantly different only in FEF.

Connectivity patterns can be interpreted using the Gaussian curve and the Yerkes–Dodson law. Up to a certain point, higher functional connectivity signifies a healthy brain with substantial cognitive potential. This is also observed when concentrating on a difficult task or compensating for temporary or chronic deficits in functioning. Deficits and disconnections in one network have been shown to cause hyperconnectivity in another compensating for losses in the disrupted network. Presumably, hyperconnectivity constitutes an observable brain response to neuronal network disturbances (48, 49). However, full compensation is characteristic only of relatively healthy brains or as an early sign of disease (50–53). Various studies suggest that the neuronal networks of individuals with disturbed functioning are both ineffective as well as inadequate due to an inability to sufficiently compensate for their deficits (53–56). Notably, only transient hyperconnectivity is considered advantageous, while prolonged hyperconnectivity can lead to detrimental effects on the human brain. Hillary and Grafman (2017) present similar observations highlighting that while hyperconnectivity is adaptive in the short term, chronic hyperconnectivity makes network hubs vulnerable to secondary pathological processes due to chronically elevated metabolic stress (48). Moreover, chronic hyperconnectivity is associated with serious progressive neuronal impairments observed in schizophrenia (57, 58). Excessive connectivity increases brain metabolism leading to higher amyloid beta (Aβ) deposition, which accelerates brain aging and destabilizes its functioning. In summary, chronic hyperconnectivity, associated with compensatory mechanisms, can be seen as a biomarker of progressive neuronal impairments (48, 59–61).

Increased FC between bilateral FEF and IPS in SZ is consistent with previous studies indicating dysconnectivity within DAN in this clinical group (22). Disrupted activity of this system corroborates with the studies linking dorsal stream abnormalities with deficits of stereopsis (62), fragmented object recognition (63), motion perception (64), and cognitive disorganization (13). Our observations of IPSs dysconnectivity corresponds with the findings that SZ patients present altered activation of posterior parietal cortex and IPS that is linked with impairments of visual working memory and perceptual organization deficits, respectively (27, 65). Our results provide further evidence of DAN dysconnectivity in SZ. The observation that SZ patients present excessive FC within DAN compared to BD patients correspond with the results of the studies evaluating attention deficits and their neuronal underpinnings in those clinical groups. Several studies have shown that SZ patients present worse performance than HC during tasks known to activate DAN (visual search and Posner cueing tasks) (26, 66), while individuals with BD obtain results comparable to those of HC (67, 68). VanMeerten et al. (2016) have shown that contrary to BD, SZ patients present abnormal early brain responses during visual search task suggesting that diminished early posterior brain responses are specifically associated with SZ neuropathology (26). Pokorny et al. (2021) have demonstrated that SZ patients present aberrant cortical connectivity of IPS during object recognition task, which is not observed in BD (27). This observation corresponds with our results indicating that excessive between IPS and FEF are observed only in SZ, while there are no significant differences in terms of those connections between the BD and HC groups. Keane et al. (2023) suggested that differences in DAN task activity between BD, SZ, and HC are so large that they might yield a candidate biomarker for differential diagnosis. Results of our study points out that resting-state DAN-FC may have similar potential of discriminating the abovementioned groups as it reveals an opposite profile of activity between BD and SZ individuals (hypo- vs. hyperconnectivity) (13). The possibility of using rs-fMRI for this purpose is promising as this technique may be used without requiring any specific task or stimulus. To the best of our knowledge, this is the first study identifying differences within DAN FC between the abovementioned clinical groups. Most of the single-scanner studies evaluating large-scale functional networks did not include DAN in their analyses (28–33). A recent study of Huang et al. (2020) evaluating transdiagnostic and illness-specific functional dysconnectivity across SZ, BD, and major depressive disorder patients did not find differences in FC within DAN between the studied group; however, an abnormal connectivity pattern between DAN and the left hippocampus has been recognized as one of the SZ-specific deficits (20). Further studies are required to establish the potential of DAN–FC to differentiate the abovementioned groups.

We are aware of several limitations of our study as follows: (a) a relatively small number of participants; (b) heterogeneity of BD patient group in terms of the disorders type (12 BD I and 18 BD II individuals)—small number of subjects in those groups did not allow us to analyze the effect of this variable on DAN–FC; (c) patients were not drug naïve, which could affect the results; however, we have shown that equivalent of the daily dose of olanzapine is not associated with FC measures, which corresponds with the results of a recent study indicating no impact of antipsychotics on connectivity strength in BD and SZ (69).

Our study shows that resting state FC within DAN may differentiate euthymic BD, remitted SZ, and healthy individuals. Divergent patterns of FC within DAN, reflected in hyperconnectivity in BD and hypoconnectivity in SZ, might yield a candidate biomarker for differential diagnosis between both conditions. More highly powered analyses are needed to confirm these possibilities. Future studies should evaluate whether resting state FC within DAN is associated with attention deficits in SZ and BD.

## Data Availability

The raw data supporting the conclusions of this article will be made available by the authors, without undue reservation.
